# Acetyl-l-carnitine (ALCAR) for the prevention of chemotherapy-induced peripheral neuropathy in patients with relapsed or refractory multiple myeloma treated with bortezomib, doxorubicin and low-dose dexamethasone: a study from the Wisconsin Oncology Network

**DOI:** 10.1007/s00280-014-2550-5

**Published:** 2014-08-29

**Authors:** Natalie Callander, Stephanie Markovina, Jens Eickhoff, Paul Hutson, Toby Campbell, Peiman Hematti, Ronald Go, Robert Hegeman, Walter Longo, Eliot Williams, Fotis Asimakopoulos, Shigeki Miyamoto

**Affiliations:** 1 University of Wisconsin Carbone Cancer Center, 600 Highland Ave, Madison, WI 53792 USA; 2 Department of Biostatistics and Medical Informatics, University of Wisconsin School of Medicine and Public Health, Madison, WI USA; 3 Gundersen-Lutheran Health System, LaCrosse, WI USA

**Keywords:** Multiple myeloma, Bortezomib, Neuropathy, Acetyl-l-carnitine, NF-kB

## Abstract

**Purpose:**

Retreatment with bortezomib (B) is often considered for patients with relapsed multiple myeloma (MM), but this strategy is hindered by uncertainty of response and emergence of B-induced peripheral neuropathy (PN). We incorporated acetyl-l-carnitine (ALCAR) to prevent PN and allow for adequate dosing. We also investigated the correlation between B-inducible NF-κB activation and response to therapy.

**Methods:**

Nineteen patients with relapsed/refractory MM received up to 8 cycles of intravenous bortezomib, doxorubicin and oral low-dose dexamethasone (BDD) to evaluate response and toxicity. Thirteen additional patients received prophylactic ALCAR (BDD-A). Patients receiving BDD-A were evaluated by FACT-GOG-TX, FACIT-Fatigue, Neuropathic Pain index (NPI) and Grooved Pegboard (GP) testing. Primary MM cells from 11 patients were tested for B-inducible NF-κB activation.

**Results:**

Seventy-six percent of subjects were refractory to previous treatment, 39 % refractory to bortezomib. Median cycles received were 5. CR + PR for the entire group were 53 % and did not differ between groups. Incidence of ≥3 PN was 32 % in the BDD group versus 15 % in the BDD-A group (*p* = ns). Patient-reported fatigue and PN measured by FACT-GOG-TX increased throughout the treatment period in the BDD-A group, although time to complete GP testing declined. In a sub-study examining constitutive bortezomib-inducible NF-κB activity in primary subject-specific MM cells, the presence of NF-κB activation correlated with lower likelihood of response.

**Conclusions:**

Addition of ALCAR to BDD did not alter the incidence or severity of PN in relapsed MM patients receiving a B-based regimen. Bortezomib-inducible NF-κB activation in patient-derived primary MM cells may be associated with poorer response.

## Introduction

Multiple myeloma is an incurable plasma cell malignancy with a course characterized by initial responsiveness to treatment, followed by the appearance of increasingly refractory disease and ultimately death due to infection, renal failure and cytopenias [1]. Until the last two decades, patients with MM had few therapeutic options after failing initial therapy with oral alkylators and steroids. Bortezomib, the first clinically approved proteasome inhibitor proved to be a powerful new agent for the treatment of MM [2]. Initial trials of single agent bortezomib for relapsed MM resulted in response rates of 30–35 % [3, 4]. However, MM patients rapidly acquire resistance to bortezomib when used as a single agent, and it is apparent that this resistance may in part be modified through combination with other existing chemotherapy agents, including steroids, alkylating agents, anthracyclines and immunomodulatory drugs [5–7]. As more experience has been gained with these combinations, it is also apparent that some patients who have previously received bortezomib can be retreated at the time of relapse with excellent results [8]. However, predicting which relapsed patients will respond to retreatment with bortezomib remains essentially a “trial and error” process.

An additional obstacle when considering retreatment of MM patients has been the rapid development of peripheral sensory and occasionally motor neuropathy that is the most frequently observed important non-hematologic toxicity of bortezomib. The exact mechanisms underlying bortezomib-induced PN remain unclear. Animal models of bortezomib-induced PN demonstrate damage to dorsal root ganglia (DRG) neuronal cell bodies including chromatolysis and accumulation of electron dense juxtanuclear cytoplasmic deposits [9]. Casafont et al. [10] demonstrated accumulation of poly (A) RNAs in nuclear granules and suggested that interference with pre-mRNA processing may be a major pathologic event in the development of PN. The incidence and severity of peripheral neuropathy (PN) is influenced by the schedule used (e.g., twice weekly versus weekly [11]), the route of administration (intravenous push versus subcutaneous [12]), and concurrent administration of other chemotherapeutic agents. In particular, combining bortezomib with anthracyclines produces some of the highest rates and severity of PN [13, 14]. The presence of certain genetic polymorphisms may also influence the incidence and severity of bortezomib-related PN [15, 16].

In an effort to ameliorate peripheral neuropathy, some investigators have suggested the addition of various agents thought to be neuroprotective at the time PN emerges. For example Richardson et al. [17] in the initial phase II study of bortezomib for subjects with newly diagnosed myeloma, offered patients gabapentin as well as other supplements at the emergence of ≥grade 1 neuropathy. Anecdotal reports of improvement of PN have also been attributed to the use of a number of supplements, including alpha lipoic acid [18], and B vitamins including pyridoxine [19] and vitamin B12 [20].

Acetyl-l-carnitine (ALCAR), another compound of interest, is an ester of l-carnitine and a critical component in mitochondrial energetics and function. ALCAR appears to act as a transport molecule for fatty acids in and out of the axonal mitochondria, where they are then utilized in energy-producing metabolic cycles. In animal models of chemotherapy-induced PN, ALCAR, administered to rats dosed with paclitaxel, vincristine, oxaliplatin, or cisplatin, decreased the incidence of allodynia (the sensation of pain in response to a normal stimuli) [21–24]. It is not yet clear whether the benefits noted from ALCAR are due to the intact ester or to the combined effect of the acetyl and l-carnitine moieties formed by hydrolysis of the parent molecule. Other attractive features of ALCAR are that it is extremely well tolerated, has no known drug interactions, comes as an oral preparation and is available without prescription as a dietary supplement. Studies in human subjects have suggested that ALCAR may be effective in treating patients with chemotherapy-induced NP in two uncontrolled, pilot trials [25, 26]. We therefore investigated the ability of ALCAR to prevent the emergence of bortezomib-induced PN in a two part pilot study using the combination of bortezomib, doxorubicin and dexamethasone in a heavily pretreated group of patients. We specifically allowed enrollment of patients refractory to the bortezomib and dexamethasone doublet. In the first half of the study patients received the three-drug combination without ALCAR to examine the response rate and toxicities. Although seemingly an active combination, we noted a high incidence of PN that affected the ability to deliver subsequent cycles of therapy. Therefore, patients accrued to the second part of the study received prophylactic ALCAR to see if the incidence and severity of PN would be reduced and to determine if response rates were maintained when ALCAR was included.

As an additional exploratory component of the study, we also sought to determine if we could predict an individual subject’s response to the BDD combination by examining the inherent NF-κB activation status of the patient’s primary myeloma cells as this transcription factor activity has been implicated in promoting survival of cancer cells. The biological mechanisms underlying primary or acquired bortezomib resistance are not known although the development of structural variants in the β5-subunit of the 20S proteasome core has been cited [27]. Previously our group has demonstrated that a subset of primary MM cells isolated from patient bone marrow samples display enhanced constitutive NF-κB activity when exposed to bortezomib and such activation in MM cell lines correlated with bortezomib-resistance in vitro [28]. We therefore sought to evaluate in a subset of patients receiving treatment on this trial whether we could correlate clinical response with this phenomenon. We hypothesized that MM cells from responding subjects would be less likely to show bortezomib enhanced NF-κB activity and that non-responders would display bortezomib-inducible NF-κB activity.

## Methods

Patients with relapsed and/or refractory MM, including those relapsing on bortezomib, were eligible. Progressive/relapsed disease was defined as an increase of >1 g/dl of monoclonal protein, or >200 mg of protein in a 24-h urine collection; those subjects who progressed thusly while on or within 60 days of treatment were deemed refractory. Patients were informed of the investigational nature of the study and signed informed consent. The study was conducted at all Wisconsin Oncology Network sites after appropriate approval by individual institutional review boards in compliance with the Declaration of Helsinki and Good Clinical Practice Guidelines. Patients with preexisting >grade 2 peripheral motor or sensory neuropathy were excluded. Minimum laboratory requirements included absolute neutrophils >1,500/mcl and platelets >100,000/mcl (unless the cytopenias were due to marrow replacement with MM), ALT and AST < 3 times the institutional upper limits, no more than 220 mg/m^2^ of previous doxorubicin exposure and left ventricular ejection fraction >40 % as determined by echocardiogram or MUGA within the previous 90 days. Prior to treatment, patients were staged with serum protein electrophoresis, 24-h urine collection, skeletal survey and bone marrow biopsy.

Treatment consisted of bortezomib (B) 1.3 mg/m^2^ on day 1, 4, 8 and 11 intravenously, doxorubicin (D) 15 mg/m^2^ on days 1 and 8 intravenously and dexamethasone (Dex) 20 mg by mouth on days 1, 4, 8 and 11 for up to 8 cycles. Prophylactic acyclovir was required to prevent varicella reactivation. Dose reductions of B and D were used for cytopenias and treatment emergent PN. Growth factor use was allowed and encouraged per investigator discretion to maintain the dosing schedule.

As part of the neuroprotection portion of the trial, subjects received the identical chemotherapy regimen but in addition received ALCAR 1.5 g by mouth twice daily. These patients answered the FACT-GOG-NTX and FACIT-Fatigue [29] questionnaires at the time of enrollment, and prior to each odd cycle. A member of the research team administered the Grooved Peg Board test [30] in duplicate. Patients in either cohort achieving a complete remission (CR) underwent a bone marrow biopsy at that time to confirm response. CR was defined as no monoclonal protein detectable by immunofixation of serum and urine as well as <5 % plasma cells in the marrow. Partial response (PR) was defined as >50 % reduction in serum monoclonal protein, minimal response (MR) between 25 and 50 % reduction and progressive disease as at least 25 % increase in serum M protein (minimum >0.5 g/dl) or 200 mg in urine protein from the lowest level, new lytic bone lesions, or hypercalcemia. Responses were evaluated every two cycles.

### Statistical analysis

Study outcomes were summarized in terms of means, standard deviations and ranges, or frequencies and percentages. Duration of response and overall survival were analyzed using the Kaplan–Meier method. GP, FACIT-Fatigue, FACT/GOG-Neurotoxicity and NPI were analyzed using linear mixed effects models with subject-specific random effects and paired t tests. An exact paired McNemar’s test was used to compare proportions between baseline and end of study assessments. All *p* values were two-tailed and *p* < 0.05 was used to define statistical significance. Data analysis was conducted using SAS software version 9.3 (SAS Institute, Cary, NC).

### NF-κB DNA-binding activity in primary MM cells

Bone marrow aspirate samples were collected from subjects at the time of enrollment in the initial BDD cohort for the inducible NF-κB analysis. Eleven aspirate samples were evaluable. Eight other subjects could not be evaluated either because a bone marrow sample was not sent for analysis by a study site or because the sample did not yield enough viable cells for analysis. CD138+ cells were sorted from aspirates and then were cultured with or without 100 nM bortezomib (predetermined concentration required for >80 % proteasome inhibition). The cells were then lysed and NF-κB DNA-binding activity was evaluated using the electrophoretic mobility shift assay (EMSA) adapted for small amounts of cellular protein [31]. The analysis of NF-κB DNA-binding activity was performed without knowledge of the patient’s response, and the data were only correlated with actual clinical data after all marrow samples had been analyzed.

### Results of BDD/BDD-A treatment

A total of 19 patients were treated with BDD and 13 patients received BDD plus ALCAR (BDD-A). Table 1 displays characteristics of all subjects. The median age of the entire cohort was 64.5 years (range 39–88), and the median number of previous regimens was 5 (range 1–8). The median time from diagnosis to enrollment on the trial was a 29.1 months (range 5.4–108.1) for the entire cohort and was not statistically different between the BDD and BDD-A groups. Nineteen (59 %) patients were previously exposed to bortezomib. Twelve (38 %) of the patients were refractory to bortezomib, and 25 (78 %) were refractory to their most recent treatment. Fifteen (47 %) patients had undergone peripheral blood stem cell transplantation. Nineteen percent of patients had diabetes, 34 % had preexisting grade 1 neuropathy, related primarily to previous thalidomide or bortezomib exposure.

**Table 1:** Patient demographics (*n* = 32)

	Mean (SD)	Median	Range
Age (years)	63.1 (11.7)	64.5	39–88
Number of previous treatments	4.9 (2.2)	5.0	1–8
Time from diagnosis to study entry (months)	37.1 (26.3)	29.1	5.4–108.1

	*N*	%
Gender		
Female	11	34
Ethnicity		
Non-hispanic	28	88
Hispanic	3	9
Unknown	1	3
Dose modifications while on study	12	37
Previous treatment with bortezomib	19	59
Refractory to previous treatment	25	78
Refractory to bortezomib	12	38
Lytic lesions	8	25
Diabetes	6	19
Preexisting neuropathy (≤grade 2)	11	34

The percentage of patients with high-risk cytogenetic karyotype or FISH, defined as 13 deletion by cytogenetics, 17p deletion, t(4:14), t (14:16) and t(14; 20) included 6/19 (32 %) subjects in the BDD group and 6/13 subjects (46 %) in the BDD-A cohort and overall 38 % of subjects.

### Response to treatment

One patient in the BDD cohort was found to be ineligible due to preexisting LFT abnormalities, and four patients experienced progressive disease during the first two cycles of therapy. Response rates of the remaining subjects were 53 % (95 % CI 36–69 %), with a CR + PR rate of 53 % (95 % CI 32–73 %) in the BDD cohort and 54 % (95 % CI 29–77 %) in the BDD-A cohort. If minimal response is included as an assessment of clinical benefit, these rates increased to 63 % (95 % CI 45–77 %) among all subjects, with 53 % (95 % CI 32–73 %) in the BDD and 77 % (95 % CI 50–92 %) in the ALCAR cohort (*p* = 0.35). These results compare favorably to previously reported results in relapsed/refractory patients receiving either bortezomib/liposomal doxorubicin or PAD therapy. Not surprisingly, the response rates observed in high-risk patients were lower, 17 % in BDD and 33 % in BDD-A, respectively.

The median number of cycles of therapy delivered was 5 in both cohorts (range 1–8). The median duration of response was 3 months in the BDD cohort versus 10 months in the BDD-A cohort (*p* = 0.097). Median overall survival rate calculated from time of enrollment for the group was 28.3 months (range 0.2–75.3+), with a median overall survival of 22.9 in the BDD cohort and 28.3 in BDD-A cohort (*p* = ns).

#### Toxicity

Patients in both cohorts experienced significant hematologic toxicity as expected in this heavily pretreated cohort. No deaths were directly attributable to the treatment regimen. Forty-two percent (8/19) in the BDD and 46 % (6/13) in the BDD-A group developed >grade 3 hematologic toxicity while on therapy, primary neutropenia and thrombocytopenia. Non-hematologic toxicity, mostly GI such as diarrhea, occurred in 41 % of the entire cohort. One subject in the BDD group developed a grade 4 infection with CMV (Table 2).

**Table 2:** Frequencies and percentages of treatment associated toxicities

	BDD (*N* = 19)		BDD-A (*N* = 13)		BDD + BDD-A (*N* = 32)
	Grade 3	Grade 4	Grade 3	Grade 4	Any grade
	*N* (%)	*N* (%)	*N* (%)	*N* (%)	*N* (%)
ANC	1 (5)	3 (16)	2 (15)	2 (15)	15 (47)
Hemoglobin	1 (5)	1 (5)	1 (8)	0 (0)	8 (25)
Platelets	0 (7)	7 (37)	1 (8)	1 (8)	18 (56)
Infection	3 (2)	2 (11)	0 (0)	3 (23)	7 (22)
Lymphopenia	1 (5)	0 (0)	0 (0)	0 (0)	1 (3)
Nausea	1 (5)	0 (0)	0 (0)	0 (0)	7 (22)
Fatigue	1 (5)	0 (0)	0 (0)	0 (0)	10 (31)
Neuropathy	6 (32)	0 (0)	2 (15)	0 (0)	20 (62)
Pain	1 (5)	0 (0)	0 (0)	0 (0)	9 (28)
Diarrhea	2 (11)	1 (5)	2 (15)	0 (0)	14 (44)

**Table 3:** GP, FACIT-fatigue, FACT/GOG-neurotoxicity and NPI scores for BDD-A patients

	Baseline (*N* = 13)	Cycle 3 (*N* = 12)		End of treatment (*N* = 10)	
	Mean (SD)	Mean (SD)	*p* value^a^	Mean (SD)	*p* value^b^
GP (Grooved Pegboard)					
Dominant (s)	99.5 (18.9)	103.7 (30.5)	0.443	103.0 (33.1)	0.711
Non-dominant	94.1 (19.2)	101.2 (22.7)	0.221	94.1 (17.7)	0.326
FACIT-fatigue	14.9 (11.0)	16.3 (7.7)	0.762	22.4 (11.2)	0.114
FACT/GOG-neurotoxicity	5.9 (5.4)	8.9 (6.7)	0.113	10.9 (10.0)	0.101
NPI					
Intense	1.8 (2.7)	3.1 (3.2)	0.438	4.5 (3.4)	0.313
Sharp	1.3 (2.4)	2.8 (3.2)	0.313	2.6 (3.4)	0.875
Hot	0.3 (0.7)	1.4 (2.4)	0.375	3.3 (3.4)	0.125
Dull	0.8 (1.5)	2.8 (3.6)	0.125	4.2 (3.0)	0.063
Cold	0.0 (0.0)	0.9 (1.8)	0.500	2.3 (3.3)	0.250
Sensitive	0.1 (0.9)	0.9 (1.5)	0.375	3.2 (3.1)	0.031*
Itchy	0.0 (0.0)	0.3 (0.5)	0.500	1.6 (3.1)	0.250
Total	4.1 (6.3)	12.0 (14.4)	0.406	21.7 (14.2)	0.047*

	*N* (%)	*N* (%)	*p* value	*N* (%)	*p* value
FACIT-fatigue < 30	12 (92)	11 (92)	0.999	8 (80)	0.980
FACIT-GOG NP > 0	8 (62)	9 (75)	0.480	6 (60)	0.999

* Statistically significant *p* < 0.05

^a^p value for evaluating changes from baseline to cycle 3

^b^p value for evaluating changes from baseline to end of treatment

#### Neurotoxicity

**Fig. 1:**
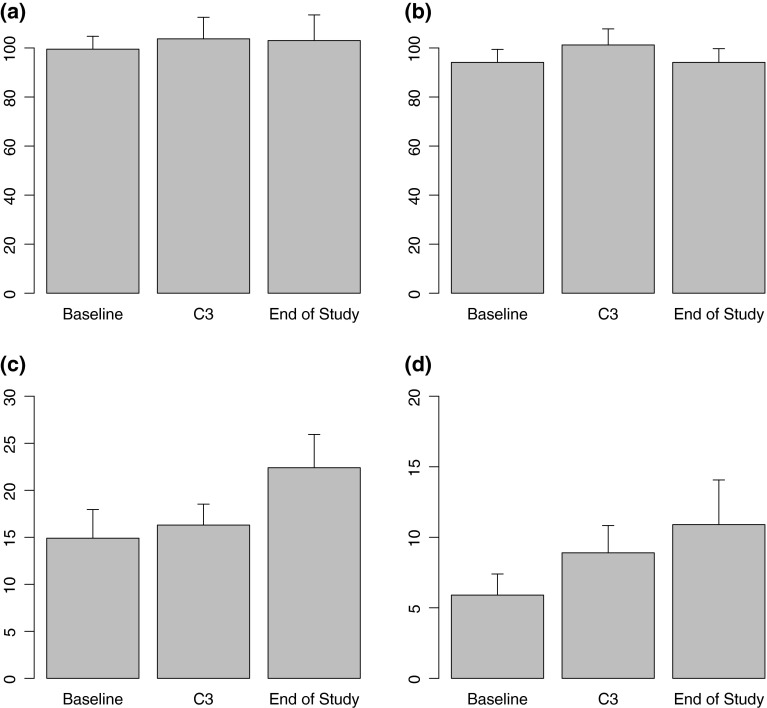
Mean and standard errors for GP, FACIT-Fatigue and FACT/GOG-Neurotoxicity scores at baseline, cycle 3 and end of study; **a** GP-dominant, **b** GP-non-dominant, **c** FACIT-Fatigue, and **d** FACIT/GOG-Neurotoxicity

**Fig. 2:**
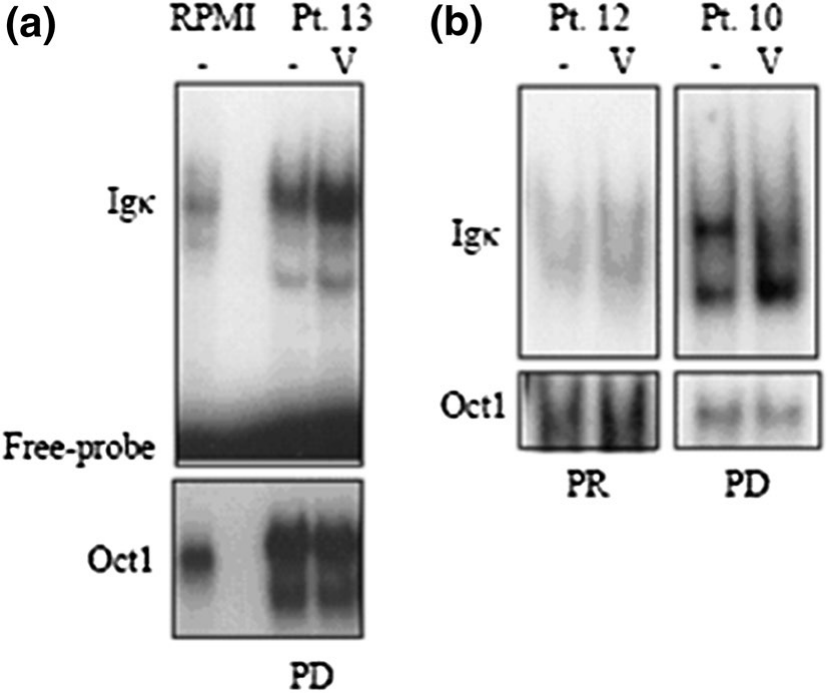
Electrophoretic mobility shift assay (EMSA) for NF-κB binding in RPMI8226 (“RPMI,” human multiple myeloma cell line) or primary CD138+ cells sorted from Pt. 10, 12 or 13 (as labeled). The NF-κB-specific band is designated “igκ” and the un-bound probe is labeled “Free probe”. Oct-1-DNA binding is used as a loading control. Lanes labeled “V” were treated with 100 nM bortezomib for 4 h prior to harvest. Pt 10 and 13 showing bortezomib-inducible NF-κB activity did not respond to BDD treatment; pt 12, with no bortezomib-inducible Nf-κB activity, achieved a partial response (PR) to BDD

As assessed by the treating physician, six of 19 (32 %) receiving BDD developed grade 3/4 neuropathy, primarily sensory with one subject experiencing grade 4 neuropathy. However, in the ALCAR cohort, 2/13 (15 %) experienced grade ≥3 neurotoxicity, with no patients experiencing treatment emergent grade 4 PN. This difference is not statistically different. Using a score of >0 on the FACT-GOG-NTX scale on questions 1–4, 8 or 9, the baseline prevalence of any subjective neuropathy among the 13 patients in the BDD-A group cohort was 62 % (8/13). We also performed a statistical analysis of BDD-A subjects comparing results at the beginning and end of therapy on the Grooved Pegboard (GP), the FACT-GOG-NTX, the FACIT-Fatigue and the NPI index (Table 3). Twelve (92 %) subjects reported significant fatigue prior to starting protocol-specified treatment using a cutoff score of <30 on the FACIT-Fatigue Scale [29]. Only 2/13 reported an improvement in fatigue by the time they terminated the treatment (*p* = 0.98). The majority of patients reported an increase in both overall fatigue and symptoms such as numbness, allodynia and tingling, as well as general discomfort in both the hands and feet over the course of the study as measured by the NPI (Figs. 1, 2), although these increases were not statistically significant.

### NF-κB assessment: prediction of response to bortezomib

A total of 11 subjects in the BDD cohort had bone marrow samples available for testing of baseline NF-κB activation status in the presence of bortezomib. Plasma cells from seven subjects did not display significant inducible NF-κB activation; of those, 5 (71 %) patients achieved a clinical response (Fig. 2). In contrast to the 4 patients with bortezomib-inducible NF-κB activity, one (25 %) patient achieved stable disease, 2 patients experienced progressive disease and one patient died during the first week of therapy due to disease. Although these patient numbers are small, this assay suggests that bortezomib-inducible activation of NF-κB may be an important marker of bortezomib resistance and could be used for treatment allocation.

## Discussion

Bortezomib is an extremely active agent in relapsed MM patients, especially when combined with steroids and other compounds such as alkylating agents. In particular, numerous reports attest to the utility of retreatment with bortezomib. We have demonstrated that the combination of doxorubicin, low-dose dexamethasone and bortezomib is associated with a high response rate of 53 % (CR and PR), even in very refractory patients. This observed response rate is similar to that reported by Palumbo et al. [32] using a different dosing schedule of bortezomib, doxorubicin and dexamethasone. They found an overall response rate in 67 % of patients, although an important difference is that in their trial, greater than 60 % of patients received the three-drug bortezomib-based combination as their first- or second-line relapse therapy. However, both preexisting and treatment emergent neuropathy continues to be an important consideration that limits long-term administration of bortezomib. We attempted to mitigate the incidence and severity of PN through the use of prophylactic acetyl-l-carnitine. Our study suggests that the addition of ALCAR did not eliminate treatment-related PN, although there appeared to be fewer cases of grade 3 or 4 neuropathy among patients receiving the prophylaxis as reported by the treating physicians. However, as measured by validated instruments such as the FACIT-GOG-NTX and the NPI index, the subjects reported increasing levels of neuropathy and continuing fatigue as they continued on study. Given the observed continued high responses to the BDD-A combination, it is clear that the inclusion of this agent in the treatment regimen did not diminish the response rate and ALCAR was very well tolerated. Major limitations of our study include the study’s small subject numbers and that we did not assess subjects receiving BDD with the same instruments, relying on PN assessment by study personnel using CTCAE criteria. Of note, in their BDD combination study, Palumbo reported only a 10 % incidence of grade 3–4 treatment emergent PN, substantially lower than the 25 % noted for our entire study. An important factor may be that the preexisting prevalence of PN in their subjects was 22 % compared to our subjects at 34 %.

Initial trials incorporating bortezomib quickly pointed to PN as an important toxicity of this drug [32, 33]. Other trials incorporating bortezomib in previously treated patients have reported similar rates of PN and grade 3–4 toxicity. Orlowski et al. [34] in the pivotal trial of pegylated doxorubicin and bortezomib reported an 80 % incidence of ≥grade 3 adverse events (AE): 36 % of patients stopped therapy due to an AE. The incidence of PN was reported as 35 % with only 4 % reported as grade 3/4. Richardson et al. [16] published data from the SUMMIT and CREST trial of bortezomib and dexamethasone in relapsed MM patients showing that 80 % of subjects either reported PN or were clinically assessed as having PN. In our much smaller study, we found that only 55 % of these heavily pretreated subjects reported significant PN but some of this difference may be due to the lower rate of exposure to thalidomide prior to enrollment (51 %) compared to subjects in those trials (72 %). Our observed rate of treatment emergent PN was also higher than that reported in either the SUMMIT [3] or CREST [4] trials. Newer methods of bortezomib dosing, such as subcutaneous or weekly administrations, appear to significantly lower the rate of PN, but it remains an important issue [11, 12, 35]. These modifications would seem the current best approach in minimizing bortezomib-related PN as the preliminary data from our small study does not support any advantage for the inclusion of ALCAR in this clinical setting. ALCAR administered intramuscularly has been shown in a randomized double-blind trial to improve retroviral therapy-induced PN [36] and in a small series of patients on RT receiving long-term oral ALCAR [37]. It is therefore conceivable that the lack of protective effect in our trial may be due to different mechanisms of neuronal injury caused by bortezomib. It is also conceivable that the incorporation of ALCAR in bortezomib containing regimens earlier in the treatment course, e.g., for newly diagnosed MM patients, might offer a protective advantage against the development of PN. However, Hershman recently reported results from a large placebo-controlled, randomized trial in which breast cancer patients receiving adjuvant taxane-based chemotherapy were assigned to ALCAR versus placebo. Their study found no improvement in patient-reported PN and actually showed an increase in PN severity in patients receiving ALCAR as measured by FACT-NTX and clinician assessment after 24 weeks of therapy [38]. Given the scope of this trial, it seems less likely that the reason we also did not observe a reduction in PN with the inclusion of ALCAR was due sample size.

We also examined the pretreatment activation status of NF-κB in primary myeloma cells obtained from bone marrow in a subset of patients. We found a correlation between clinically relevant bortezomib-resistance and bortezomib-inducible NF-κB activation. Despite the use of what is considered an active regimen, there was a distinctly lower response rate among those patients whose primary myeloma cells displayed this characteristic. This bortezomib-inducible activity was found both in subjects with previous exposure to B and in one B-naive subject who failed to respond to BDD. These interesting results raise the possibility of using such an assay at the time of staging bone marrow biopsy to help determine optimal therapy. However, the assay as conducted here requires large numbers of MM cells and we are attempting to modify the assay to allow for use of smaller aliquots of primary MM cells [39]. In the future, we hope this technology could be incorporated in real time to determine the most effective therapeutic options for newly diagnosed and relapsed patients.

## References

[1] Kyle R, Rajkumar SV (2006). Multiple Myeloma. Blood.

[2] Hideshima T, Richardson P, Chauhan D (2001). The proteasome inhibitor PS-341 inhibits growth, induces apoptosis and overcomes drug resistance in human multiple myeloma cells. Cancer Res.

[3] Richardson PG, Barlogie B, Berenson J (2003). A phase 2 study of bortezomib in relapsed, refractory myeloma. N Engl J Med.

[4] Jagannath S, Barlogie B, Berenson J (2004). A phase 2 study of two doses of bortezomib in relapsed or refractory myeloma. Br J Haematol.

[5] Berenson JR, Yang HH, Sadler K (2006). A phase 1/2 trial assessing bortezomib and melphalan combination therapy for relapsed or refractory multiple myeloma. J Clin Oncol.

[6] Ciolli S, Leoni F, Gigli F (2006). Low dose velcade, thalidomide and dexamethasone (LD-VTD): an effective regimen for relapsed and refractory myeloma. Leuk Lym.

[7] Palumbo A, Ambrosini MT, Benevolo G (2007). Bortezomib, melphalan, prednisone and thalidomide for relapsed multiple myeloma. Blood.

[8] Petrucci MT, Giraldo P, Corradini P (2013). A prospective international phase 2 study of bortezomib retreatment in patients with relapsed multiple myeloma. Br J Haematol.

[9] Cavaletti G, Gilardini A, Canta A (2007). Bortezomib induced peripheral neurotoxicity: a neurophysiological and pathological study in the rat. Exp Neurol.

[10] Casafont I, Berciano M, Lafarga M (2010). Bortezomib induces the formation of nuclear poly (A) RNA granules enriched in Sam68 and PABN1 in sensory ganglia neurons. Neurotox Res.

[11] Bringhen S, Larocca A, Rossi D (2010). Efficacy and safety of once weekly bortezomib in multiple myeloma patients. Blood.

[12] Moreau P, Pylpenko H, Grosicki S (2011). Subcutaneous versus intravenous bortezomib in patients with relapsed multiple myeloma. A randomized, phase 3 non inferiority trial. Lancet Oncol.

[13] Orlowski RZ, Nagler A, Sonneveld P (2007). Randomized phase II study of pegylated liposomal doxorubicin plus bortezomib compared with bortezomib alone in relapsed or refractory multiple myeloma: combination therapy improves time to progression. J Clin Oncol.

[14] Chang JE, Peterson C, Choi S (2011). Vcr-CVAD induction chemotherapy followed by maintenance rituximab in mantle cell NHL. Br J Haematol.

[15] Corthals SL, Kuiper R, Johnson DC (2011). Genetic factors underlying the risk of bortezomib induced peripheral neuropathy in multiple myeloma patients. Hematologica.

[16] Broyl A, Cornthals S, Jongen J (2010). Mechanisms of peripheral neuropathy associated with bortezomib and vincristine in patients with newly diagnosed multiple myeloma: a prospective analysis of data from HOVON-65/GMMG-HD4 trial. Lancet Oncol.

[17] Richardson PG, Xie W, Mitsiades C (2009). Single-agent bortezomib in previously untreated multiple myeloma: efficacy, characterization of peripheral neuropathy and molecular correlations with response and neuropathy. J Clin Oncol.

[18] Gedlicka C, Kornek GV, Schmid K (2003). Amelioration of docetaxel/cisplatin induced polyneuropathy by α-lipoic acid. Ann Oncol.

[19] Garg MB, Ackland SP (2011). Pyridoxine to protect from oxaliplatin-induced neurotoxicity without compromising antitumor effect. Cancer Chemother Pharmacol.

[20] Medina-Satillan R, Morales-Franco G, Espinoza-Raya J (2004). Treatment of diabetic neuropathic pain with gabapentin alone or in combination with vitamin B complex. Preliminary Results. Proc West Pharmacol Soc.

[21] Ghirardi O, Vertechy M, Vesci L (2005). Chemotherapy-induced allodynia: neuroprotective effect of acetyl-L-carnitine. In vivo.

[22] Pisano C, Pratesi G, Laccabue D (2003). Paclitaxel and cisplatin-induced neurotoxicity: a protective role of acetyl-L-carnitine. Clin Cancer.

[23] Flatters SJL, Xiao WH, Bennett GJ (2005). Acetyl-L-carnitine prevents and reduces paclitaxel-induced painful peripheral neuropathy. Neurosci Lett.

[24] Ghirardi O, Lo Guidice P, Pisano C (2005). Acetyl-L-carnitine prevents and reverts experimental chronic neurotoxicity induced by oxaliplatin, without altering its antitumor properties. Anticancer Res.

[25] Bianchi G, Vitali G, Caraceni A (2005). Symptomatic and neurophysiological responses of paclitaxel- or cisplatin-induced neuropathy to oral acetyl-L-carnitine. Eur J Cancer.

[26] Maestri A, De Pasquale Ceratti A, Cundari S (2005). A pilot study on the effect of acetyl-L-Carnitine in paclitaxel and cisplatin-induced peripheral neuropathy. Tumori.

[27] Oerlemans R, Franke NE (2008). Molecular basis of bortezomib resistance: proteasome subunit beta5 (PSMBA5) mutation and overexpression of PSMB5 protein. Blood.

[28] Markovina S, Callander NS, O’Connor S, Kim J, Werndli J, Raschko M, Leith C, Kahl B, Kim K, Miyamoto S (2008). Bortezomib-resistant nuclear factor-κB activity in myeloma cells. Mol Cancer Res.

[29] Cella D, Yount S, Sorensen M (2005). Validation of the Functional Assessment of Chronic Illness Therapy Fatigue Scale relative to other instrumentation in patients with rheumatic arthritis. J Rheumatol.

[30] Ruff RM, Parker SB (1993). Gender- and age-specific changes in motor speed and eye-hand coordination in adults: normative values for the finger tapping and grooved pegboard tests. Percep Mot Skills.

[31] O’Connor S, Shumway SD, Amarna LI, Hayes CE, Miyamoto S (2004). Regulation of constitutive p50/c-Rel activity via proteasome inhibitor-resistant Iκβα degradation in B cells. Mol Cell Biol.

[32] Palumbo A, Gay F, Bringhen S (2008). Bortezomib, doxorubicin and dexamethasone in advanced multiple myeloma. Ann Oncol.

[33] Aghahanian C, Dizon DS, Sabbatini P (2005). Phase I trial of bortezomib and carboplatin in recurrent ovarian or primary peritoneal cancer. J Clin Oncol.

[34] Orlowski RZ, Voorhees PM, Garcia RA (2005). Phase I trial of the proteasome inhibitor bortezomib and pegylated liposomal doxorubicin in patients with advanced hematologic malignancies. Blood.

[35] Moore S, Atwal S, Sachchithanantham S (2013). Weekly intravenous bortezomib is effective and well tolerated in relapsed/refractory myeloma. Eur J Haematol.

[36] Youle M, Osio M (2007). A double blind, parallel group, placebo-controlled multicentre trial of acetyl-L-carnitine in the symptomatic treatment of antiretroviral toxic neuropathy in patients with HIV-1 infection. HIV Med.

[37] Herzmann C, Johnson A, Youle M (2005). Long term affect of acetyl-L-carnitine for antiretroviral toxic neuropathy. HIV Clin Trials.

[38] Hershman DL, Unger JM, Crew KD (2013). Randomized double-blind placebo-controlled trial of acetyl-L-carnitine for the prevention of taxane-induced neuropathy in women undergoing adjuvant breast cancer therapy. J Clin Oncol.

[39] Young EM, Pak C, Kahl BS, Yang DT, Callander NS, Miyamoto S, Beebe D (2012). Microscale functional cytomics for studying hematologic cancers. Blood.

